# Modeling and nonlinear predictive control of solar thermal systems in district heating

**DOI:** 10.1016/j.heliyon.2024.e31027

**Published:** 2024-05-14

**Authors:** Jan Lorenz Svensen, Hjörleifur G. Bergsteinsson, Henrik Madsen

**Affiliations:** Department of Applied Mathematics and Computer Science, Technical University of Denmark, Richard Petersens Plads 324, 2800 Kongens Lyngby, Denmark

**Keywords:** Data-driven modeling, MPC, Solar thermal, District heating systems

## Abstract

This study presents data-driven modeling and nonlinear model predictive control of solar thermal plants in district heating, for the purpose of operation optimization. The study considers the efficient operation of a solar thermal plant in Hillerød, Denmark. A dynamic model is estimated as a system of stochastic differential equations using grey-box modeling and real-world data. The presented nonlinear model predictive controller design is based on repeated trajectory linearization and the dynamic model. Several objective designs are considered, e.g., maximizing energy or temperature. The study provides simulations for analyzing the model fitness and controller performances. The model is shown to fit the daytime operation of the plant. The designed controllers are shown to improve efficiency, increasing the transported energy up to 28%.

## Highlights

• Data-driven modelling of a solar thermal system.
• Design of Nonlinear Model Predictive Controllers (NMPC) for the solar thermal system.
• Performance analysis of the application of NMPC.
• Comparison with historical performances.

## 1 Introduction

The efficient and sustainable operation of a District Heating System (DHS) depends on the available options for heat production, the demands, and the layout of the network. DHS is often centered on a single thermal plant or combined heat and power plant. Some DHS is supplemented by heat production from renewable sources, e.g. electric heat pumps or Solar Thermal Plants (STP). An example is the DHS in Hillerød, Denmark, which incorporates an STP. The option of additional heat sources provides DHSs with the flexibility to optimize their efficiency [1]. For example through an electricity-heat production tradeoff or by reducing fuel consumption.

Efficient production and distribution of heat throughout the DHS requires the knowledge of when and where heat is available and is needed. Thus forecasting the operation of the heat sources is a necessity for optimizing the entire DHS. This requires models and operation predictions, as well as access to environmental forecasts.

In this work, we will focus on the STP heat source. STPs provide the DHS with green heat in the daytime, by converting solar energy into thermal heat. The heat production of STPs can operationally be considered independent from the DHS. This work considers the optimal operation and control of STPs, as these can be considered external inputs to the DHS. As predictions of the operation are required, the Model Predictive Control strategy (MPC) is considered to provide the heat forecasts needed by the DHS. MPC is considered for the built-in predictive capabilities and it can incorporate system limitations into the model, such as pump flow limits.

### 1.1 Related work

Predicting the operation of STP requires a model describing the dynamics, a control strategy for the STP, and forecasts of the weather; e.g. solar irradiation and air temperature.

Several STP models have been proposed in the literature. [2] proposed a model for the solar collectors using the theoretical power output and instantaneous solar irradiance for a case study in Høje Taastrup, Denmark. [3] considered a case study in Latvia, proposing steady-state models for evaluating efficiency and demand. [4] considered steady-state models for STP and desalination plants in DHS. [5] proposed a model for a solar HVAC system, giving hybrid dynamic formulations for solar collectors, heat exchangers and storage tanks. [6] considered the size-wise design of solar-based DHS using the local efficiency. [7] presented methods for modelling solar collectors using SDEs and grey-box modelling. [8] suggested a correlation model for forecasting solar radiation with hourly resolution accounting for stochastic features. [9] proposed the use of splines to model the solar gain factor using a grey-box SDE modelling to account for the sun's position.

In the literature, MPC is described as a mature control strategy [10], [11], [12] applied to several fields [13]; e.g., solar tanks [14], sewage systems [15], [16], and robotics [17]. In DHS research, MPC has been used for lowering supply temperature [18], peak-shaving of supply power [19], utilized in decentralized control of the district heating [20], and for solar thermal plants [5], [21], [22]. In [21] a linear MPC is designed for the STP, which is described as a nonlinear lumped Ordinary Differential Equation model with a quadratic air temperature term, while in [5], a storage tank is included, while no quadratic air term is used. In [22] an MPC focusing on minimizing the pumping power is proposed for a DHS with an STP, using logic-based objectives based on the level of stored energy. [23] considered the tracking piece-wise constant references in the STP using robust linear MPC based on a time-invariant linearized model. [24] proposed using gain-scheduling dual mode MPC for an STP, utilizing LTI models linearized to local operation points.

### 1.2 Contributions

Compared to the existing literature on STP control, we design the nonlinear MPC (NMPC) utilizing repeated trajectory linearization. This incorporates the nonlinearities of the STP in the predictions instead of basing the predictions on linearizations of fixed operation points. Thus, the predictions account for changing nonlinear effects in the forecasted operation.

We consider the case of an STP in a DHS located near Hillerød, Denmark. For the modeling of the STP, we take inspiration from the literature. The model structure is inspired by [7], with the application of the solar-heat gain model of [9]. Thus the paper formulates a continuous-time model for the STP using stochastic differential equations (SDEs) in a grey-box formulation based on real-world data from the STP.

Several design objectives are considered for the control, e.g., maximizing temperature or transferred energy to DHS. The current controller design at the STP in Hillerød is used to evaluate the developed controllers. The performances are compared to both historical data and simulation. The optimality of the current control strategy is discussed in view of MPC performances.

The paper is organized as follows. Section 2 presents the specific STP considered. Section 3 provides the modeling of the plant. Section 4 describes the controllers, while Section 5 presents the simulation results and Section 6 discusses the results. Section 7 provides the conclusions.

## 2 Solar thermal plant

This section presents the STP considered in this study and the available measurements. An STP transforms solar energy into thermal heat and transports it to the receiving DHS. The STP consists of solar collectors heated by solar irradiation and cooled by the surrounding air and a controlled flow through the collectors. The performance depends on the sun's place in the sky (the angle of irradiation) and the cloudiness of the sky (the irradiation strength, and ratio between direct and diffuse radiation).

### 2.1 Hillerød solar thermal plant

**Figure 1:**
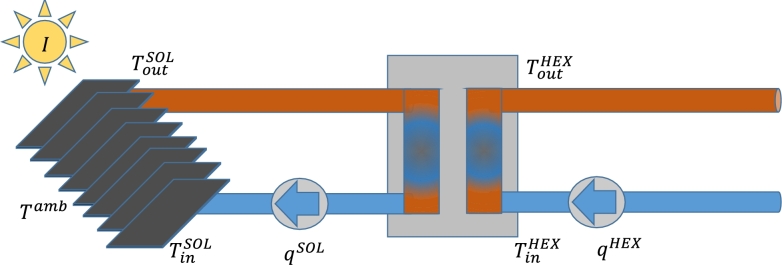
Schematic view of a section of the Hillerød district heating system. Notation corresponds to measurements in Table 1.

The considered STP is located in Hillerød, a city north of Copenhagen, Denmark, operated by *Hillerød Forsyning*. It supplies Hillerød and surrounding towns with water and heating. Moreover, the considered plant's role is to supply a single area within the Hillerød district heating network. With 3007 m^2^ of solar panels, it is designed such it can supply all required heating in the area during summer. The plant consists of flat plate collectors and is connected to the DHS through a heat exchanger; decoupling the temperature of the DHS with the fluctuating temperature of the STP. The system operates without the usage of storage elements [25]. Fig. 1 provides a schematic overview of the plant operation; it shows the collectors, the plant pipes, the heat exchanger, and the two pumps that circulate the fluid through the heat exchange at the STP and the DHS side.

### 2.2 Data

The measurements used were collected over 8 days at the STP between 03-06-2021 to 10-06-2021. The local weather information was provided by a climate station at *Hillerød Forsyning*. The measured variables are listed in Table 1, along with the corresponding notation used in the following sections. As the different measurements have different sampling times, the measurements used have been re-sampled to 5 min. sampling, to align them at sampling times.

**Table 1:** Available measurements from the plant and the weather station. HEX is the Heat Exchanger, and STP is the Solar Thermal Plant.

Measurement	Variable	Unit
Heat at HEX	$P_{t}^{\mathrm{HEX}}$	kW
Heat at STP	$P_{t}^{\mathrm{SOL}}$	kW
Supply Temp. at HEX	$T_{out,t}^{\mathrm{HEX}}$	^∘^*C*
Supply Temp. at STP	$T_{out,t}^{\mathrm{SOL}}$	^∘^*C*
Return Temp. at STP	$T_{in,t}^{\mathrm{SOL}}$	^∘^*C*
Return Temp. at HEX	$T_{in,t}^{\mathrm{HEX}}$	^∘^*C*
Pump flow at STP	$q_{t}^{SOL}$	$\frac{m^{3}}{s}$
Pump flow at HEX	$q_{t}^{HEX}$	$\frac{m^{3}}{s}$
Ambient Air Temperature	$T_{t}^{\mathrm{amb}}$	^∘^*C*
Global Solar Intensity	*I*_*t*_	$\frac{W}{m^{2}}$

## 3 Solar thermal plant modelling

For optimizing or predicting the behavior of the plant, a model is a necessity. In this study, we apply grey-box modeling to formulate a dynamic model, by incorporating physical knowledge of the system and system identification based on data from the STP. As we consider a real STP, its dynamics operate in continuous time.

For the states of the model, we consider the temperatures exiting, $T_{out,t}^{\mathrm{SOL}}$, and entering, $T_{in,t}^{\mathrm{SOL}}$, the solar panels and exiting, $T_{out,t}^{\mathrm{HEX}}$, the heat exchanger, while the control is given by the two flows ($q_{t}^{SOL}$, $q_{t}^{HEX}$). As any real measurement is uncertain to some degree, we find it reasonable to assume the system is stochastic. Thus the dynamics of the model are based on stochastic differential equations (SDE). The SDEs in this paper will be based on the following continuous-discrete time stochastic state space form [26],

(1) $$dx_{t}=f(x_{t},u_{t},\theta )dt+g(\theta )d\omega_{t}$$

(2) $$y_{t_{k}}=h(x_{t_{k}})+e_{t_{k}}$$

where the states, $x_{t}$, evolve continuously as determined by the drift term, *f*, and diffusion term, *g*. The observation function, *h*, relates measurements and states. $u_{t}$ is external inputs, *θ* is the system parameters, $y_{k}$ is the discrete measurements. The drift term, accounts for most of the known dynamics, while the diffusion term relates to unaccounted and unknown dynamics as well as noise, like uncertainty in the input variables. The stochastics can be described by constant diffusion terms and measurement noise variance.

For formulating the structure of the drift term, the literature contains some examples of dynamical modeling of STPs. [2] suggested a heat flow as a quadratic-linear sum of the difference between ambient air and STP mean temperature with a linear irradiance term. [21] and [5] suggest basing the quadratic-linear sum on the difference between ambient air and outlet STP temperature, with an additional term for the inlet-outlet STP temperature difference. [7] suggested an SDE model, linear to the temperatures.

The model identification procedure in this study will not be stated explicitly however the presented model is inspired by [7]. The initial model is

(3) $$dT_{out,t}^{\mathrm{SOL}}=\frac{1}{mC}(U_{1}(T_{t}^{\mathrm{amb}}-\frac{T_{out,t}^{\mathrm{SOL}}+T_{in,t}^{\mathrm{SOL}}}{2})+\mu I_{t}+c_{p}q_{t}^{SOL}(T_{in,t}^{\mathrm{SOL}}-T_{out,t}^{\mathrm{SOL}}))dt+e_{1}^{p_{1}}d\omega_{t}^{\left(1\right)}$$

which was derived from the result in Perers [27].

As the interest is in the transport of heat from the solar collectors, the model has been formulated with $T_{out,t}^{\mathrm{SOL}}$ as the state. [9] demonstrated that the solar gain is a time-varying process due to the angle of the solar beams hitting the collectors changes over the day between the sun rises and sets, and that using splines instead of a constant *μ* resulted in significant improvements when modeling the outlet temperature of the solar collectors. Therefore the approach using a spline function is applied in the model.

As the heat from the solar collectors is used to heat the flow to the DHS through a heat exchanger, the model is extended with a heat exchanger model. The dynamics of the heat exchanger are assumed to be counter-current flow plate heat exchanger as described in [28].

Finally, the proposed overall system to model the stochastic dynamics of a solar thermal plant and heat exchanger is

(4) $$\begin{array}{cc} dT_{out,t}^{\mathrm{SOL}}= & \frac{1}{mC}(U_{1}(T_{t}^{\mathrm{amb}}-\frac{T_{out,t}^{\mathrm{SOL}}+T_{in,t}^{\mathrm{SOL}}}{2})+S(t,\alpha )I_{t}+c_{p}q_{t}^{SOL}(T_{in,t}^{\mathrm{SOL}}-T_{out,t}^{\mathrm{SOL}})+U_{2}(T_{in,t}^{\mathrm{HEX}}-T_{out,t}^{\mathrm{SOL}}))dt+e_{1}^{p_{1}}d\omega_{t}^{\left(1\right)} \end{array}$$

(5) $$T_{\Delta }=\frac{T_{out,t}^{\mathrm{SOL}}+T_{in,t}^{\mathrm{SOL}}}{2}-\frac{T_{out,t}^{\mathrm{HEX}}+T_{in,t}^{\mathrm{HEX}}}{2}$$

(6) $$\begin{array}{cc} dT_{in,t}^{\mathrm{SOL}}= & \frac{1}{mC}(c_{p}q_{t}^{SOL}(T_{out,t}^{\mathrm{SOL}}-T_{in,t}^{\mathrm{SOL}})-U^{HEX}T_{\Delta })dt+e^{p_{2}}d\omega_{t}^{\left(2\right)} \end{array}$$

(7) $$\begin{array}{cc} dT_{out,t}^{\mathrm{HEX}}= & \frac{1}{mC^{HEX}}(c_{p}q_{t}^{HEX}(T_{in,t}^{\mathrm{HEX}}-T_{out,t}^{\mathrm{HEX}})+U^{HEX}T_{\Delta })dt+e^{p_{3}}d\omega_{t}^{\left(3\right)}. \end{array}$$

$T_{t}^{\mathrm{amb}}$ is the ambient air temperature. $T_{in,t}^{\mathrm{HEX}}$ is the return temperature from the district heating flowing into the heat exchanger (right side). *mC* and $mC^{HEX}$ are thermal capacitances. $U_{1}$, $U_{2}$, and $U^{HEX}$ are heat transmission coefficients. $I_{t}$ is the solar intensity and S(t,α) is the effective size of the solar panels hit by solar radiation, described by the splines, *bs*:

(8) $$S(t,\alpha )=\sum_{i=1}^{4}\alpha_{i}bs_{i,t}$$

The transporting medium is water (with the specific heat constant as $c_{p}=4.2\frac{J}{g^{\circ }C}$), and the state measurements are described by

(9) $$Y_{1}\in N(T_{out,t}^{\mathrm{SOL}},\sigma_{1}^{2})$$

(10) $$Y_{2}\in N(T_{in,t}^{\mathrm{SOL}},\sigma_{2}^{2})$$

(11) $$Y_{3}\in N(T_{out,t}^{\mathrm{HEX}},\sigma_{3}^{2})$$

### 3.1 System identification

**Figure 2:**
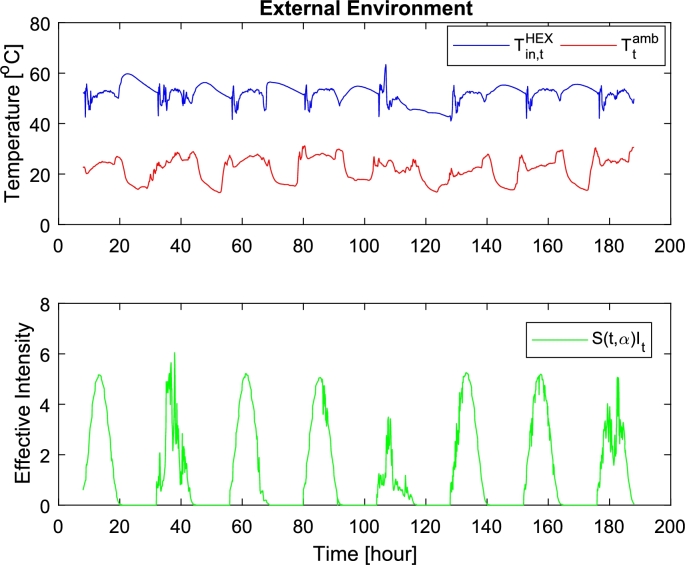
Environment's inputs: showing the driving inputs over an 8-day period, Up) the return and air temperatures, Down) the effective solar intensity, illustrating both smooth and erratic days.

For the identification of the parameters in the SDE, the estimation is done using a maximum likelihood method and the first 12 hours of daytime data in the 8-day data set. Fig. 2 shows the external temperatures and solar intensity during the 8 days. The nighttime data is excluded from the training set, as the data has an unexpected heat source during the night, possibly due to an unmeasured backflow leakage from the DHS [25]. Practically we are using the ctsm-r package in R [29] with a known initial state (at t=0). The estimated parameters and the standard deviation of their uncertainty can be found in Table 2.

**Table 2:** Parameters, estimates, and their standard deviation (sd) for Eq. (4)-(7), with *α*_*i*_ being the splines coefficients in Eq. (8) and *ϵ*_*i*_ representing the variances $\sigma_{i}^{2}$ in Eq. (9)-(11) by $\epsilon_{i}=ln(\sigma_{i}^{2})$.

par	$T_{out,0}^{SOL}$	$T_{in,0}^{SOL}$	$T_{out,0}^{HEX}$	*α*_1_	*α*_2_	*α*_3_	*α*_4_	*mC*	*mC*^*HEX*^
estimate	48.1	44.3	47.54	0.0026	0.0081	0.0052	0.0016	0.545	170.6519
sd	NA	NA	NA	6e-04	8e-04	9e-04	0.0013	0.8001	40.9957

par	*ϵ*_1_	*ϵ*_2_	*ϵ*_3_	*p*_1_	*p*_2_	*p*_3_	*U*_1_	*U*_2_	*U*^*HEX*^
estimate	-8.1827	-9.6273	2.0654	0.2307	-2.9704	0.1501	0.013	0.0441	0.5267
sd	12.014	9.7965	0.231	0.8211	0.0611	0.6807	0.0041	0.0117	0.0181

**Figure 3:**
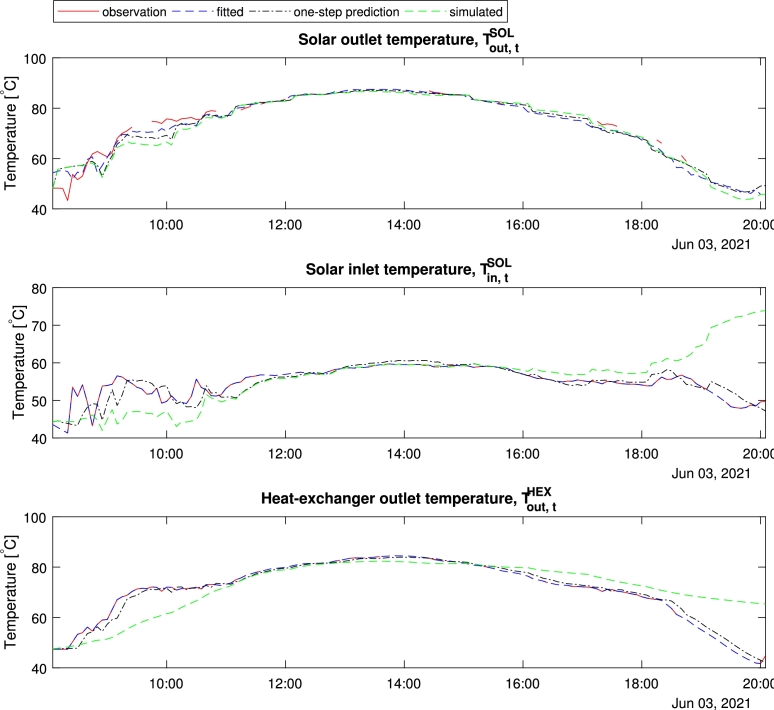
Fitting results of 1-day simulation; given in time of the day.

Fig. 3 shows the historical and simulated system temperatures for the 12 hours used for the training. We can see the simulation captures the general behavior of the system dynamics. In the early and late hours discrepancies are observed, possibly due to less training in the conditions with low solar intensity.

**Figure 4:**
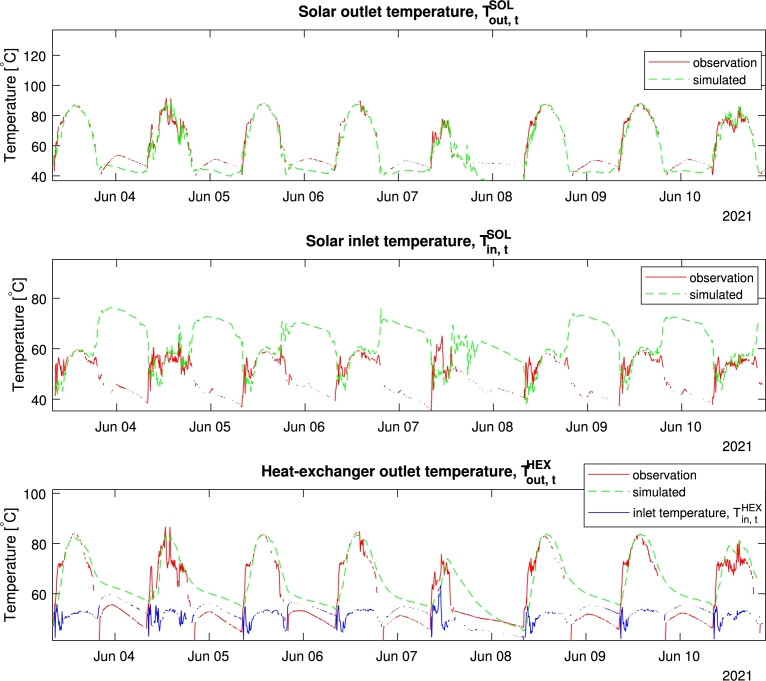
8-days simulation.

Fig. 4 shows the simulation of all 8 days. We can see the fit and discrepancy repeats for each signal; capturing the general dynamics during the daytime, with clear discrepancies during the night. These discrepancies illustrate unaccounted dynamics such as the mentioned backflow, when no flow should occur. Despite the night discrepancies, we can observe that the model quickly realigns itself as soon as the day starts. Given that operationally the system is only of interest during daytime, the model is still applicable for the purpose of prediction and control in those periods.

## 4 Control

In this section, we will design a couple of nonlinear MPCs to operate the district heating subsystem. The different MPCs will be designed with slightly different objectives; maximizing energy transfer, temperature, or both, or temperature reference tracking.

### 4.1 Stability of the system

When designing an MPC, the stability of the dynamics is important to ensure the desired behavior. We will therefore provide a brief analysis of the stability.

For a continuous-time linear system, a system is stable if its state matrix A has negative eigenvalues, Eig(A)<0, while for nonlinear systems this is more complicated [30]. Given the diffusion term is constant, stability is determined by the deterministic part of the system. If we consider our inputs to the system to be time-varying components, we can analyze the nonlinear system as a linear time-variant system on the form:

(12) $${\overset{˙}{T}}_{t}=A_{t}T_{t}+b_{t}$$

(13) $$A_{t}=\left[\begin{array}{ccc} -\frac{\frac{U_{1}}{2}+U_{2}+c_{p}q_{t}^{SOL}}{mC} & -\frac{\frac{U_{1}}{2}-c_{p}Q_{t}}{mC} & 0\\ -\frac{\frac{U^{HEX}}{2}-c_{p}q_{t}^{SOL}}{mC} & -\frac{\frac{U^{HEX}}{2}+c_{p}q_{t}^{SOL}}{mC} & \frac{U^{HEX}}{2mC}\\ \frac{U^{HEX}}{2mC^{HEX}} & \frac{U^{HEX}}{2mC^{HEX}} & -\frac{\frac{U^{HEX}}{2}+c_{p}Q_{t}^{HEX}}{mC^{HEX}} \end{array}\right]$$

(14) $$b_{t}=\left[\begin{array}{c} \frac{U_{1}T_{t}^{amb}+S(\alpha )I+U_{2}T_{in,t}^{HEX}}{mC}\\ \frac{U^{HEX}T_{in,t}^{HEX}}{2mC}\\ \frac{c_{p}q_{t}^{HEX}-\frac{1}{2}U^{HEX}}{mC^{HEX}}T_{in,t}^{HEX} \end{array}\right]$$

Based on the structure of $A_{t}$, we can conclude that the disturbances do not affect A's eigenvalues; only the flows do. If we then consider the workspace of the flows, the theoretical highest eigenvalue of each state becomes

(15) max(Re(Eig(A)))=[−0.800710−6−0.0789−0.4049]

and hence we can conclude that $A_{t}$ is stable at any time independently of the flows; with the almost-zero worst-case eigenvalue corresponding to the zero-flow case. Thus we can conclude that at any point the system is stable in the sense it is converting towards some stationary state:

(16) $$T_{\infty }=-A_{\infty }^{-1}b_{\infty }.$$

### 4.2 Low-level controller

The control of the physical system consists of two layers; two low-level PI-controllers directly regulating the flows based on the output from a higher layer controller [25]. The installed PI-controller has the coefficients shown in Table 3. This controller can be added to our system as:

(17a) $${\overset{˙}{q}}_{pid,t}^{SOL}=\frac{1}{1+K_{p1}}{\overset{˜}{q}}_{t}^{SOL}-\frac{K_{i1}}{1+K_{p1}}q_{pid,t}^{SOL}$$

(17b) $$q_{t}^{SOL}=\frac{K_{i1}}{K_{p1}+1}q_{pid,t}^{SOL}+\frac{K_{p1}}{K_{p1}+1}{\overset{˜}{q}}_{t}^{SOL}$$

(17c) $${\overset{˙}{q}}_{pid,t}^{HEX}=\frac{1}{1+K_{p2}}{\overset{˜}{q}}_{t}^{HEX}-\frac{K_{i2}}{1+K_{p2}}q_{pid,t}^{HEX}$$

(17d) $$q_{t}^{HEX}=\frac{K_{p2}}{K_{p2}+1}{\overset{˜}{q}}_{t}^{HEX}+\frac{K_{i2}}{K_{p2}+1}q_{pid,t}^{HEX}$$

**Table 3:** Parameters of PI-controllers.

par	*K*_*p*1_	*K*_*i*1_	*K*_*p*2_	*K*_*i*2_
value	1	0.0333	0.3	0.0055

Stability-wise, we can see that (17a) and (17c) are scalar linear equations with negative state coefficients, so the added states are asymptotically stable.

### 4.3 MPC design

**Figure 5:**
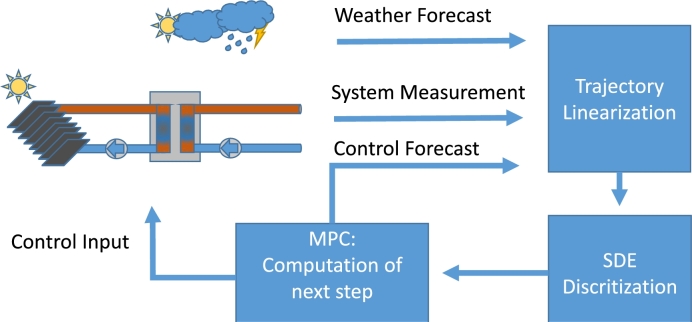
Graphical representation.

As the system and model are nonlinear SDEs, the design of an NMPC includes how to address the nonlinearities and the stochastic parts. In this work, the NMPC will be deterministic, so the controller will operate at the nominal case. For the nonlinearity, the NMPC will be constructed as a linear MPC utilizing re-linearization at each time step [31], as shown by Algorithm 1 and illustrated in Fig. 5. The linearizations will be trajectory-based, utilizing the “forecasted” inputs and flows to generate each linearization points in the horizon. This approach was chosen to account for the occasional erratic behavior of the inputs, e.g. the solar intensity, see Fig. 2.

**Algorithm 1:**
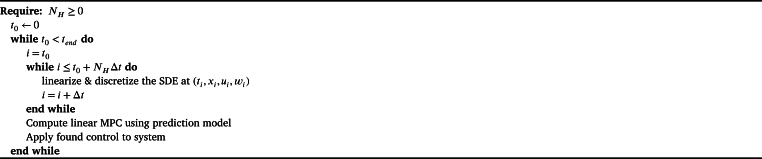
Nonlinear MPC.

The MPC designs will utilize 5 min. sampling in its discretization, and prediction horizons, $N_{H}$, of 24 samples, corresponding to 2 hours. The length was chosen such that the horizon has sufficient coverage to account for the coming changes in inputs, without risking obtaining too large an optimization program.

#### 4.3.1 Cost function

The design of MPCs depends on the definition of optimality as described by the cost function. For our MPC designs, we utilize the quadratic structure:

(18) $$J_{t}=\min_{u}\sum_{i=0}^{N_{H}}||z_{t+i}|{|}_{H_{z}}^{2}+g_{z}^{T}z_{t+i}+g_{u}^{T}u_{t+i}+||\Delta u_{t+i}|{|}_{H_{\Delta u}}^{2}$$

where $z_{i}$ is the objectives of the MPCs outlined in Table 4 together with the corresponding weight matrices. The used notations are; $||z|{|}_{H}^{2}$ for the weighted quadratic norm $z^{T}Hz$, ${\text{1}}_{n}$ notes a size n vector of ones, $I_{n}$ notes the n×n identity matrix, and $diag(x_{1},x_{2})$ notes the diagonal matrix with $x_{1}$ and $x_{2}$ on the diagonal. The weights in each controller are tuned manually together, by visual inspection of the temperatures and flows. The energy transfer objective $P_{t}^{HEX}$ is defined by:

(19) $$P_{t}^{HEX}=c_{p}(T_{out,t}^{HEX}-T_{in,t}^{HEX})q_{t}^{HEX}$$

The objectives were chosen based on the operational goals of Hillerød Forsygning [25]; namely maximizing energy transfer from the STP to the DHS, as well as having high temperatures suitable for mixing with the district heating. This was interpreted as maximizing energy on the DHS side, and/or maximizing the delivered temperature or keeping it at a certain temperature reference, chosen to be 80C∘.

**Table 4:** Weights used in the objective of the MPC variants.

MPC variant	Objective *z*	*H*_*z*_	*g*_*z*_	*g*_*u*_	*H*_Δ*u*_
Energy	$P_{t}^{HEX}$	0	−100	$1600{\text{1}}_{2}$	10^6^*I*_2_
Energy+Max Temperature	$\left\{T_{out,t}^{HEX},P_{t}^{HEX}\right\}$	0	[−7.25,−100]^*T*^	$5000{\text{1}}_{2}$	10^6^*I*_2_
Temperature Reference-tracking	$T_{out,t}^{HEX}$	200	0	$100{\text{1}}_{2}$	10^6^*diag*(1,10)
Max Temperature	$T_{out,t}^{HEX}$	0	−100	$100{\text{1}}_{2}$	10*I*_2_

#### 4.3.2 Constraints

In MPC, we can include constraints on variables, allowing for the physical and operational limits to be included. For the four MPC versions described in Table 4, we are utilizing the same constrain set:

(20) $$\left[\begin{array}{c} T_{out,t}^{SOL}\\ T_{in,t}^{SOL}\\ T_{out,t}^{HEX} \end{array}\right]\leq \left[\begin{array}{c} 95\\ 90\\ 90 \end{array}\right],\;0\leq \left[\begin{array}{c} q_{t}^{SOL}\\ q_{t}^{HEX} \end{array}\right]\leq \left[\begin{array}{c} \frac{113}{3600}\\ \frac{102}{3600} \end{array}\right],$$

(21) $$\left[\begin{array}{c} \left|\Delta q_{t}^{SOL}\right|\\ \left|\Delta q_{t}^{HEX}\right| \end{array}\right]\leq 0.01$$

where the temperature is given in C∘ and flow in $m^{3}/s$.

The Energy-Temperature MPC was additionally implemented in a mixed-integer(MI) version with the inclusion of a logic condition on the flow $q_{t}^{HEX}$:

(22) $$T_{out,t}^{SOL}\leq 56\Rightarrow q_{t}^{HEX}=0$$

to ensure a minimum of available heat when transferring to the district heating. The specific condition, where chosen as it is part of the current rules used by the utility company [25]. Using the Mixed logical dynamical approach [32], the logic can be included in the constraints:

(23) $$\left[\begin{array}{c} T_{out,t}^{SOL}\\ T_{in,t}^{SOL}\\ T_{out,t}^{HEX} \end{array}\right]\leq \left[\begin{array}{c} 95\\ 90\\ 90 \end{array}\right],\;\left[\begin{array}{c} \left|\Delta q_{t}^{SOL}\right|\\ \left|\Delta q_{t}^{HEX}\right| \end{array}\right]\leq 0.03,$$

(24) $$\;0\leq \left[\begin{array}{c} q_{t}^{SOL}\\ q_{t}^{HEX} \end{array}\right]\leq \left[\begin{array}{c} \frac{113}{3600}\\ \frac{102}{3600}\delta_{t} \end{array}\right],\;\delta_{t}\in \{0,1\},$$

(25) $$\left(1-\delta_{t})(-156)\leq T_{out,t}^{SOL}-56\leq \delta_{t}(95-56\right)$$

where $\delta_{t}$ is an added logic variable to describe the condition.

The complete formulation of the MPC design discussed is given by (26g), with the model written in its discrete linearized form.

(26a) $$J_{t}=\min_{u}\sum_{i=0}^{N_{H}}\phi_{k+i}^{T}H\phi_{k+i}+g^{T}\phi_{k+i},$$

(26b) $$H=\left[\begin{array}{ccc} H_{z} & 0 & 0\\ 0 & 0 & 0\\ 0 & 0 & H_{\Delta u} \end{array}\right],\;g=\left[\begin{array}{c} g_{z}\\ g_{u}\\ 0 \end{array}\right],\;\phi =\left[\begin{array}{c} z\\ u\\ \Delta u \end{array}\right],$$

(26c) $${\left[\begin{array}{c} T\\ q_{pid} \end{array}\right]}_{k+1}=A_{k}{\left[\begin{array}{c} T\\ q_{pid} \end{array}\right]}_{k}+B_{k}{\left[\begin{array}{c} {\overset{˜}{q}}^{SOL}\\ {\overset{˜}{q}}^{HEX} \end{array}\right]}_{k}+G_{k},$$

(26d) $${\left[\begin{array}{c} z\\ q^{SOL}\\ q^{HEX} \end{array}\right]}_{k}=C_{k}{\left[\begin{array}{c} T\\ q_{pid} \end{array}\right]}_{k}+D_{k}{\left[\begin{array}{c} {\overset{˜}{q}}^{SOL}\\ {\overset{˜}{q}}^{HEX} \end{array}\right]}_{k}+F_{k},$$

(26e) $$\Delta q_{k}=q_{k}-q_{k-1},\;T_{k}\leq T_{max},$$

(26f) $$0\leq \left[\begin{array}{c} q_{k}^{SOL}\\ q_{k}^{HEX} \end{array}\right]\leq q_{max},\;\left[\begin{array}{c} \left|\Delta q_{k}^{SOL}\right|\\ \left|\Delta q_{k}^{HEX}\right| \end{array}\right]\leq \Delta q_{max},$$

(26g) $$u_{k}=\left[\begin{array}{c} {\overset{˜}{q}}_{k}^{SOL}\\ {\overset{˜}{q}}_{k}^{HEX} \end{array}\right],\;T=\left[\begin{array}{c} T_{out,k}^{SOL}\\ T_{in,k}^{SOL}\\ T_{out,k}^{HEX} \end{array}\right],$$

where ${}_{max}$ indicates the upper bound. $A_{k}$, $B_{k}$, $C_{k}$, $D_{k}$, $G_{k}$, and $F_{k}$ are the matrices of the repeated trajectory linearization.

## 5 Simulations

For the evaluation of the controls, simulations over all 8 days in the data set are used. The model in (7) is used as a substitute for the physical system, with the PID controller in (17a), (17b), (17c), (17d) acting as a low-level controller. The historical inputs are used as the forecast for the MPCs. Each tested controller is run with a 5 minute sampling, while the system is run in continuous time with zero-order hold on the inputs. The system's uncontrolled inputs are shown in Fig. 2, showing how the 8-days vary. The state measurements in the controls are assumed deterministic so that the controllers have ideal conditions for the evaluation.

In the following analysis, we evaluate the performance visually and numerically using KPIs considering the mean performance of the SDE model. The mean performance provides a suitable comparison, limiting the effect of stochastic drift in the analysis. We use the performance of the physical system as a baseline and relate it to the simulated performance of the MPCs. The SDEs and controls are computed using MATLAB. The used KPIs are based on the operation during daytime, which is taken as 7-21 o'clock. Table 5 shows the results of the KPIs used for the evaluations: 1) The total energy transferred at the DHS side; 2) The transferred temperature at the DHS side, and the time spent at the reference temperature, $80^{\circ }C$; 3) The amount of changes in flow usage and total amount of water pumped through the system, indicators of pump weir and energy usage; and 4) the worst-case computation time of each controller.

**Table 5:** Daytime performance comparison with the performance of the physical system as the baseline. Energy and temperature are shown as deviations from history, while flow change, volume, and computation time are in absolute values. The controllers are noted by SIM for the simulated historical control input, MT for maximizing temperature MPC, P for energy-based MPC, TR for temperature-tracking MPC, PT for energy-temperature-based MPC, and MIPT for mixed integer energy-temperature-based MPC.

Controller	Sim	MT	P	TR	PT	MIPT
Total energy [MWh]	4.2174	-18.1483	28.4660^⁎⁎^	-18.9922	28.1010	25.7647
Total Energy [%]	4.2577	-18.3220	28.7384^⁎⁎^	-19.1739	28.3699	26.0112

Temp ref. [%]	22.6143	44.3329	-29.4511	73.6158^⁎⁎^	-13.5616	-5.0047
Temp Increase [%]	5.3386	29.6771^⁎⁎^	-7.6254	23.0995	-3.7039	-1.5695

flow changes [*m*^3^/*s*]	2.1701^⁎^	3.4298	1.5960^⁎⁎^	2.3941	1.8141	1.9808
moved water [*m*^3^]	3656.5^⁎^	1872.9^⁎⁎^	7175.9	2171.6	6204.9	5587.3

Worst-case Time [*s*]	0.0623	1.5499	2.2679	1.4025	1.2684^⁎⁎^	29.4415

⁎ historical values. ⁎⁎ best performing MPC controller for the given KPI (row).

### 5.1 Flow control

**Figure 6:**
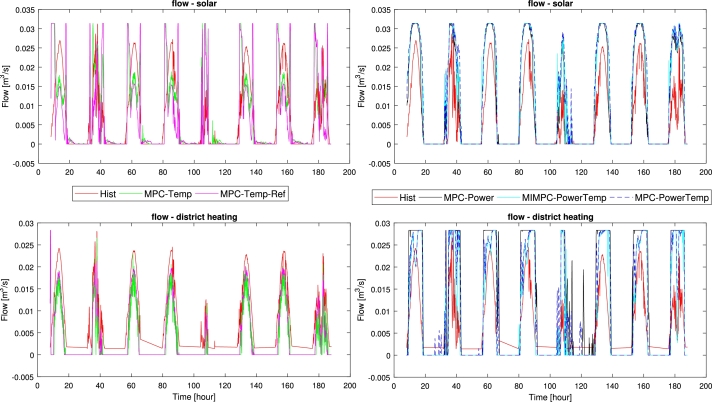
Flows: Up) pump flow of the solar side of HEX; Down) pump flow of the DHS side of HEX. Illustrating how the historic and designed controllers differ from each other in control approach over an 8-day period.

Fig. 6 shows the flows of the different controllers, with a clear difference between day and night periods. We observe that the energy-based MPCs in general utilize larger flows with smooth changes, moving upwards twice the reference volume. The temperature-based MPCs tend to lower flows, moving down to half the volume. Though the flows are more fluctuating with large changes, e.g. they have large flow periods at the beginning and end of each day, with rapid drops. During night-time, the logic-based energy-temperature MPC keeps the flows at zero, while the other energy-based MPCs have a few sporadic flows. As it generally follows the energy-temperature MPC during the daytime, this indicates the logic can generally be excluded in design considerations. From Table 5, we can observe that the energy-based MPCs use less change in pump operation, matching their smooth changes in Fig. 6. Similarly, we observe the volumes moved by the tempe-rature-based MPCs are lower than energy-based MPCs, matching the observed lower flows with short bursts of high flows. This indicates less wear on the pump for the energy-based MPCs, while the temperature-based controllers do less work, thus saving energy usage.

### 5.2 Temperature

In Table 5, the delivered temperature is shown in percentages of increase from historic performance and successful reference-tracking. As expected the temperature-based MPCs greatly improved on the temperature delivered to the DHS; showing a 29.7% improvement on raising the temperature for the Maximizing-Temperature MPC. The Temperature-tracking MPC improved time spent at the reference by 73.6%. For the energy-based MPCs, we can observe the temperatures are decreased, though only around 1−7%. This indicates a slight trade-off in temperature for increasing energy transfer.

**Figure 7:**
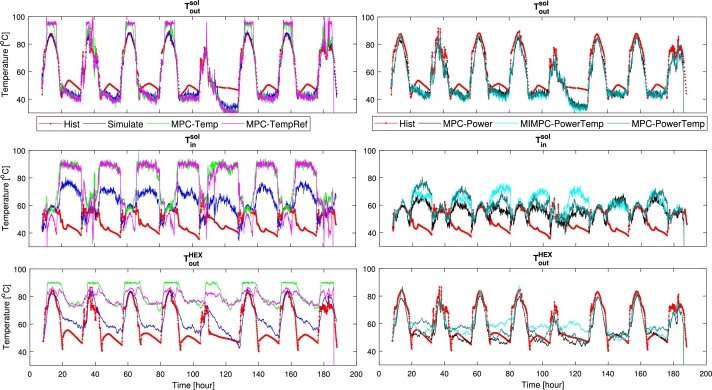
Temperature: Up) Temperature coming out of solar collectors; Mid) Temperature going into solar collectors; Down) Temperature coming out of HEX (DHS side).

Looking at Fig. 7, we observe similar results, with the temperature-based MPCs reaching temperatures much higher than the other controllers, while the energy-based controllers stay in the vicinity and behave as the historical controller, though a little lower.

### 5.3 Energy

**Figure 8:**
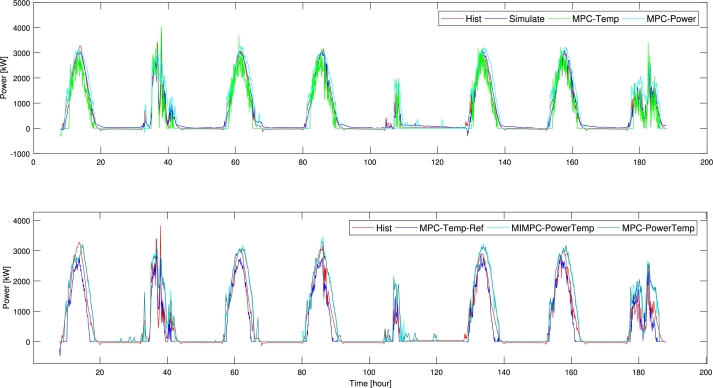
Power: the MPCs transferred energy rate to the district heating, displayed alongside the historical power. Up) simulated control, Max. Temperature MPC and Energy MPC Down) Temperature-Reference MPC, Energy+Temperature MPC, and Mixed-Integer Energy+Temperature MPC.

From Table 5, we can observe that the energy-based MPCs transfer around 28 MWh of extra energy or an improvement of 28%. For temperature-based MPCs, the energy transfers are reduced by around 18.5 MWh. Fig. 8 shows similar results, we observe that the curves of the temperature-based MPCs are below the historic curve, and more fluctuating. The energy-based MPCs give a more smooth curve, generally lying above the historic curve.

### 5.4 The model and the physical system

To evaluate the model, we compare the simulation performance using the historical flows with the historical performance, Table 5 shows the comparison (Sim). We observe a slight difference in the temperature and energy performances. The disparity gives slightly increased values across the KPIs, possibly due to the validation discrepancy in section 3. Thus we can conclude that in practice, the performance results in the above analyses should be expected to be smaller than currently observed when considering the physical system.

### 5.5 Computation

As stated, we utilized a sampling time of 5 min. In Table 5, the observed worst-case computation time for a single optimization is given. For all controllers, the computation times are significantly below the sampling time of 5 min.; generally around 1-2 seconds in worst-case. An exception is the Mixed-integer MPC, the worst computation time is 29.44 seconds, being more than 10 times slower than the second slowest.

## 6 Discussion

Based on the above analysis of the KPIs and visual performances, it is clear that none of the MPC strategies are dominating over every analysis. The maximizing energy or temperature strategies provide the best improvements on their specific KPIs. The energy-temp-based MPC appears to be the best strategy overall, it improves energy transfer close to that of the energy-based MPC, while the temperature percentage loss is only half.

Visually comparing the energy-temperature-based MPC and the historical performance, we observe that the physical system, in general, operates in the vicinity of the energy-temperature-based MPC. Typically, the physical system reaches slightly higher temperatures with similar maximum power levels, though it has steeper slopes, giving the MPC energy improvement of 28.1%. Thus the historical control can be considered a sub-optimal strategy.

The above analysis and control were taken over the mean of the stochastic simulations. Thus the stochastic nature might drift the system outside the feasible region of the constraints, making the MPC infeasible. For the considered strategies, the maximizing temperature MPC was most affected, given its extreme-driven objective. To address the stochastic nature, stochastic or robust MPC formulations could be applied.

To account for parameter drift, adaptive parameter estimation can be used for online model updates; possibly improving the discrepancy in early-late hours. This will be needed for long-term real-life operations. The used 8-day dataset where considered to be long enough to demonstrate the results, and short enough that any potential drift should be negligible; not needing an adaptive approach.

## 7 Conclusion

In this paper, Nonlinear Model Predictive Controllers (NMPC) were developed along with a data-driven model of a solar thermal plant (STP). As a case study, real-world data from a district heating system in Hillrød, Denmark were used. The STP model was estimated using grey-box modeling, and the NMPCs were formulated using repeated trajectory linearization. Performance simulations of each MPC design were presented. The performance of the NMPCs was evaluated by comparisons to the historical performance. The estimated model was shown to represent the STP for daytime operations. The NMPCs were shown to provide performance improvements for their definition of optimality. Overall, an NMPC optimizing both energy and temperature was found to provide the best performance, with a 28.1% improvement in transferred energy.

## Nomenclature

**Abbreviations:**

DHS - District Heating Systems

HVAC - Heating, Ventilation and Air-Conditioning

KPI - Key Performance Index

LTI - Linear Time-Invariant

LTV - Linear Time-Variant

MPC - Model Predictive Control

NMPC - Nonlinear MPC

ODE - Ordinary Differential Equation

SDE - Stochastic Differential Equation

STP - Solar Thermal plant

**Physics:**

*t* - time

*q* - Flow

*T* - Temperature

*P* - Energy transfer

$c_{p}$ - Heat capacity

*mC* - thermal capacitance

*U* - Heat transmission coefficient

**Math:**

Δ - difference/change

N - Gaussian Distribution

**Superscript:**

*amb* - Ambient

*HEX* - Heat exchanger

*SOL* - Solar thermal plant

## CRediT authorship contribution statement

**Jan Lorenz Svensen:** Writing – original draft, Validation, Methodology, Formal analysis, Conceptualization. **Hjörleifur G. Bergsteinsson:** Writing – review & editing, Visualization, Validation, Methodology, Investigation, Formal analysis, Data curation, Conceptualization. **Henrik Madsen:** Writing – review & editing, Supervision, Resources, Project administration, Funding acquisition, Conceptualization.

## Declaration of Competing Interest

The authors declare that they have no known competing financial interests or personal relationships that could have appeared to influence the work reported in this paper.

## Data Availability

Has data associated with your study been deposited into a publicly available repository? No, data will be made available on request.
